# Enhanced Antioxidant, Anti-Aging, Anti-Tyrosinase, and Anti-Inflammatory Properties of *Vanda coerulea* Griff. Ex Lindl. Protocorm through Elicitations with Chitosan

**DOI:** 10.3390/plants13131770

**Published:** 2024-06-26

**Authors:** Piyatida Amnuaykan, Saranya Juntrapirom, Watchara Kanjanakawinkul, Wantida Chaiyana

**Affiliations:** 1 Department of Pharmaceutical Sciences, Faculty of Pharmacy, Chiang Mai University, Chiang Mai 50200, Thailand; piyatida.chou@gmail.com; 2 Chulabhorn Royal Pharmaceutical Manufacturing Facilities by Chulabhorn Royal Academy, Chon Buri 20180, Thailand; saranya.jun@cra.ac.th (S.J.); watchara.kan@cra.ac.th (W.K.); 3 Center of Excellence in Pharmaceutical Nanotechnology, Faculty of Pharmacy, Chiang Mai University, Chiang Mai 50200, Thailand; 4 Multidisciplinary and Interdisciplinary School, Chiang Mai University, Chiang Mai 50200, Thailand

**Keywords:** *Vanda coerulea*, blue vanda, antioxidant, anti-skin wrinkle, whitening, callus, protocorm, elicitor, chitosan, cosmeceutical

## Abstract

This study aimed to investigate the effects of elicitors on *Vanda coerulea* Griff. Ex Lindl. protocorms to enhance bioactive compound production and evaluate their biological activities relevant to cosmeceutical applications. The protocorms were developed from the callus treated with different elicitors, including 6-benzylaminopurine (BA), methyl jasmonate (MeJA), and chitosan. Both the adult plant and protocorms were extracted by maceration in 80% methanol and investigated for their chemical compositions using high-performance liquid chromatography. The extracts were evaluated for antioxidant, anti-collagenase, anti-elastase, and anti-tyrosinase activities. In addition, anti-inflammatory properties were assessed using a real-time polymerase chain reaction. The irritation potency was evaluated using the hen’s egg test-chorioallantoic membrane test. The findings revealed that protocorms treated with BA and chitosan developed a greener color, while those treated with MeJA exhibited a distinct darker coloration. Elicitation with BA and chitosan resulted in protocorms with comparable or higher levels of syringic acid, rutin, and quercin compared with the adult plant, with rutin being the most prominent identified compound. Furthermore, rutin was reported as the compound responsible for all biological activities. The chitosan-treated protocorm extract exhibited potent inhibition against oxidation, collagenase, elastase, tyrosinase, and inflammatory cytokines, along with a nonirritating effect, making it a promising candidate for cosmeceutical applications.

## 1. Introduction

The rising demand for cosmeceutical products that integrate botanical-based ingredients has spurred extensive research in the beauty and skincare industry [1]. The pursuit of natural, sustainable, and plant-derived components has become essential in the development of effective and environmentally responsible cosmetics [2]. Among the potential sources of such bioactive compounds, *Vanda coerulea* Griff. ex Lindl. (Blue Vanda), an elegant orchid species indigenous to Southeast Asia, has garnered significant interest for its unique botanical attributes and traditional medicinal significance. In Ayurvedic formulations, *Vanda* spp. has been used to treat conditions such as rheumatic pain, ear infections, and nervous system disorders [3]. The bioactive compounds derived from *Vanda* species have been evaluated for numerous pharmacological activities, including neuroprotective, anti-aging, antimicrobial, anti-inflammatory, antioxidant, membrane-stabilizing, wound-healing, and hepatoprotective activities [3]. Additionally, *V. coerulea* has gained significant recognition for its remarkable contributions to anti-skin aging, promoting cellular and tissue longevity and revitalizing skin hydration and radiance [4]. These effects are attributed to the direct actions of its extract on skin cells or its ability to stimulate the enzymatic machinery involved in skin aging and related factors [4].

However, the limitations of traditional plant cultivation, characterized by the scarcity of certain botanical species and extended growth cycles, present significant challenges to the sustainable and timely utilization of valuable plant-derived compounds. Therefore, cell culture techniques stand as a promising and transformative solution, enabling efficient propagation, controlled cultivation, and large-scale production of rare and precious plant materials, particularly *V. coerulea* [5]. Currently, researchers and companies can expedite advances in drug development, nutraceuticals, and cosmeceuticals by harnessing the potential of cell culture while also supporting environmentally responsible methods that align with global sustainability goals. As the scientific community endeavors to address the complexities of biodiversity conservation and resource management, cell culture emerges as a pivotal tool, offering unparalleled opportunities for advancing botanical science and sustainable utilization of plant resources [6]. In conjunction with plantlet culture, plant cell culture techniques present a viable alternative to address the aforementioned issues [7,8]. Cultivating plant cells under controlled and favorable conditions capitalizes on their totipotency and rapid multiplication, leading to the production of secondary metabolites akin to those present in the mother plants [9,10]. Generally, the growth of orchids involves a critical phase wherein the orchid embryo matures into a protocorm before progressing into a plantlet [11]. This developmental process is continuous and facilitated by appropriate in vitro conditions, which maintain the embryogenic properties of protocorm. In medicinal contexts, the significance of protocorms lies in their potential for controlled production of bioactive compounds within orchids [12]. Harnessing this capability enables efficient cultivation of orchids for medicinal purposes, thus advancing research and development in pharmaceutical applications derived from orchid species [11]. This innovative approach offers the distinct advantage of obtaining potential secondary metabolites without necessitating the cultivation and harvest of the entire plant, thereby promoting sustainable and resource-efficient practices in drug development, nutraceuticals, and cosmeceutical research.

The utilization of protocorm culture techniques holds promise for producing biologically active compounds [13], with the key determinant of success being the identification and optimization of conditions and elicitors for optimal production. There are various elicitors that can be employed in plant cell cultures to stimulate the production of secondary metabolites, which often play crucial roles in plant defense mechanisms against environmental stressors or pathogens [14]. Elicitors, which are substances or external stimuli that trigger specific biochemical pathways in cells leading to the synthesis of bioactive compounds, can be classified into two main categories, including biotic and abiotic elicitors [14]. Biotic elicitors include substances produced by pathogens or other organisms, such as fungi or bacteria, that trigger plant defense responses, whereas abiotic elicitors are nonliving factors like physical stress (e.g., mechanical injury), chemical compounds (e.g., jasmonic acid, salicylic acid), or even environmental factors (e.g., light, temperature, humidity) that can induce the production of secondary metabolites [15,16]. The choice of elicitor depends on the specific plant species, the desired secondary metabolites, and the application in which they will be used.

Therefore, the present study aimed to cultivate *V. coerulea* protocorms utilizing various elicitors, including 6-benzylaminopurine (BA), methyl jasmonate (MeJA), and chitosan or poly-D-glucosamine. Additionally, the extracts from the *V. coerulea* protocorms and its adult plant were investigated for their phytochemical composition and capacity for cosmeceutical properties, including antioxidant, anti-aging, anti-tyrosinase, and anti-inflammatory effects. Furthermore, the irritation potency and cytotoxic effects of the extracts were also assessed.

## 2. Materials and Methods

### 2.1. Chemical Materials

Chitosan, BA, MeJA, collagenase from *Clostridium histolyticum* (EC 3.4.23.3), porcine pancreatic elastase (EC 3.4.21.36), tyrosinase from mushroom (EC 1.14.18.1), N-[3-(2-furyl) acryloyl]-Leu-Gly-Pro-Ala (FALGPA), Trizma^®^ hydrochloride buffer solution (Tris-HCl buffer), N-succinyl-(Ala)3-p-nitroanilide (SANA), phosphate buffered saline, L-3,4 dihydroxyphenylalanine (L-DOPA), epigallocatechin gallate (EGCG), kojic acid, gallic acid, chlorogenic acid, caffeic acid, ellagic acid, rosmarinic acid, catechin, quercetin, rutin, dexamethasone (DEX), 3-(4,5-dimethylthiazol-2-yl)-2,5-diphenyltetrazolium bromide (MTT), lipopolysaccharides from *Escherichia coli* (LPS), Gamborg’s B5 media, thidiazuron (TDZ), tricine, dimethyl sulfoxide (DMSO), sodium lauryl sulfate (SLS), and formic acid were analytical grades purchased from Sigma-Aldrich (St. Louis, MO, USA). TRIZOL^®^ reagent was purchased from Invitrogen (Carlsbad, CA, USA). TURBO DNA-free™ treatment kit was purchased from Ambion (Austin, TX, USA). The iScript™ cDNA Synthesis Kit was purchased from Bio-Rad Laboratories Inc. (Hemel Hempstead, UK). SYBR^®^ green was purchased from Eurogentec Ltd. (Southampton, UK). Methanol was analytical grade and acetonitrile was high-performance liquid chromatography (HPLC) grade purchased from Labscan (Dublin, Ireland). Sodium chloride (NaCl) and calcium chloride (CaCl_2_) were analytical grades purchased from KemAus™ (Western, Australia).

### 2.2. Adult V. coerulea Plants

Adult *V. coerulea* plants, aged 8 months, were acquired from the plant orchid farm at Maejo University in Chiang Mai, Thailand. The voucher specimen number A050 has been deposited at the Biotech Bangkok Herbarium in Bangkok, Thailand. The whole plant was washed with DI water and dried at an ambient temperature. The plants were cut into small pieces and dried in a hot-air oven (Memmert, Schwabach, Germany) set at 50 °C for a duration of 2 days. The dried *V. coerulea* plants were ground into powder using a Moulinex^®^ blender (Moulinex SA, Bagnolet, France) and stored in a well-closed container until further experiments.

### 2.3. V. coerulea Protocorm

*V. coerulea* callus, obtained from the plant tissue culture laboratory at Maejo University, Chiang Mai, Thailand, was a subculture in a B5 liquid medium enriched with 3 µM TDZ. The culture flask was subjected to a rotation of 90 rpm at a temperature of 25 °C, with a photoperiod lasting 16 h per day, and a light intensity set at 50 μmol/m^2^s. After a duration of four weeks, the callus was collected and used for protocorm production. Various elicitor treatments were administered, including BA, MeJA, and chitosan. In brief, 0.1 g of callus was placed in a 100 mL bottle containing 50 mL media, each supplemented with varying concentrations of different elicitors, including 3 µM of BA, 10 µM of MeJA [17], and 2 µM of chitosan [18]. Each culture was subjected to an additional four weeks of cultivation on a rotary shaker set at 90 rpm, maintaining a temperature of 25 °C, with a photoperiod of 16 h per day, and a light intensity set at 50 μmol/m^2^s. The control group comprised a cell suspension culture devoid of any interventions. Subsequently, the cultured protocorms were thoroughly rinsed with distilled water and dried using a hot-air oven (Memmert, Schwabach, Germany) set at 50 °C for a duration of 2 days. The dried protocorms were ground into powder using a Moulinex^®^ blender (Moulinex SA, Bagnolet, France). The dried powder of each protocorm was stored in a well-closed container until further experiments.

### 2.4. Extraction of V. coerulea Adult Plant and Protocorm

The *V. coerulea* plant and protocorms were extracted by maceration in methanol, following the previous method described by Giri et al. (2012) [17]. In brief, 100 mg of the dried *V. coerulea* plant and protocorm powder were macerated in 10 mL of 80% *v*/*v* methanol at room temperature with continuous stirring for 12 h. Subsequently, the resulting macerate underwent a 10 min sonication and was centrifuged at 8000 rpm for 10 min. The collected supernatant was filtered through a 0.45 µm Millipore filter (Millipore, Bedford, MA, USA). The extracts were stored in a well-closed container until further experiments.

### 2.5. Determination of Chemical Compositions of V. coerulea Plant and Protocorm Extracts Using High-Performance Liquid Chromatography (HPLC)

The chemical compositions of extracts derived from adult *V. coerulea* plants and their protocorms were examined using an HPLC technique. In brief, a Shimadzu HPLC system equipped with a UV detector set at 280 nm was employed, utilizing an Inertsil ODS-4 column (4.6 × 250 mm, 5 µm particle size) as a stationary phase. The analysis involved a mobile phase gradient consisting of acetonitrile (phase A) and a 0.1% *v*/*v* formic acid aqueous solution (phase B), operated at a flow rate of 1 mL/min. The elution gradient commenced with a 10% *v*/*v* of phase A for 10 min; then, phase A increased linearly to 20% *v*/*v* within 15 min; then, it increased further to 40% *v*/*v* within 5 min, followed by a transition to 60% *v*/*v* within 5 min; and it ultimately returned to 10% *v*/*v* within 5 min. Each extract was filtered through a 0.45 µm Millipore filter (Millipore, Bedford, MA, USA) prior to the HPLC injection with a volume of 20 µL. Various standard compounds, including kojic acid, gallic acid, chlorogenic acid, caffeic acid, ellagic acid, rosmarinic acid, catechin, quercetin, and rutin, were also injected. The content of each compound presented in the extracts from the *V. coerulea* adult plants and its protocorms was determined using the respective standard curve for each standard compound. The analysis was performed in triplicate.

### 2.6. Determination of Biological Activities of V. coerulea Adult Plant and Protocorm Extracts

#### 2.6.1. Antioxidant Activities

1. 2,2-Diphenyl-1-picrylhydrazyl (DPPH) Radical Scavenging Assay

The capability of the extracts derived from the adult *V. coerulea* plant and its protocorms to scavenge DPPH radicals was evaluated through a DPPH assay, which was modified from the methods described by Brem et al. (2004) [19]. Initially, 20 µL of the sample solution was combined with 180 µL of a DPPH solution, prepared by dissolving 167 mM DPPH in DI water. After incubating the resulting mixture in darkness at room temperature for 30 min, the absorbance was measured at 520 nm using a multimode microplate reader (BMG Labtech GmbH, Ortenberg, Germany). The findings were then expressed as the percentage of DPPH inhibition, calculated according to the subsequent equation:

DPPH inhibition (%) = [(a − b)/a] × 100,(1)

where a is the UV absorbance of the combination without a sample, while b is the UV absorbance of the combination with a sample. Various concentrations of each extract were evaluated, and their DPPH inhibitions were plotted against their logarithmic concentrations to generate dose–response curves. The IC_50_ values were calculated from the curves using GraphPad Prism (version 8.0.2, GraphPad Software, Boston, MA, USA). L-ascorbic acid was used as the positive control. The analysis was performed in triplicate.

2. Ferric-Reducing Antioxidant Power (FRAP) Assay

The capability of the extracts derived from the adult *V. coerulea* plant and its protocorms to reduce ferric was evaluated through a FRAP assay, which was modified from the methods described by Saeio et al. (2011) [20]. Initially, 20 µL of the sample solution was combined with 180 µL of the FRAP solution, prepared by mixing 0.3 M acetate buffer pH 3.6, 10 mM TPTZ in 40 mM HCl solution and 20 mM ferric chloride solution. After incubating the resulting mixture at room temperature for 5 min, the absorbance was measured at 595 nm using a multimode microplate reader (BMG Labtech GmbH, Ortenberg, Germany). The findings were then expressed as the percentage of ferric reducing ability, calculated according to the subsequent equation:

Ferric reducing abilities (%) = [(a − b)/a] × 100,(2)

where a is the UV absorbance of the combination without a sample, while b is the UV absorbance of the combination with a sample. Various concentrations of each extract were evaluated, and their ferric reducing abilities were plotted against their logarithmic concentrations to generate dose–response curves. The IC_50_ values were calculated from the curves using GraphPad Prism (version 8.0.2, GraphPad Software, Boston, MA, USA). L-ascorbic acid was used as the positive control. The analysis was performed in triplicate.

#### 2.6.2. Anti-Skin Aging Activities

3. Matrix metalloproteinase-1 (MMP-1) inhibition

The capability of the extracts derived from the adult *V. coerulea* plant and its protocorms to inhibit the MMP-1 activities was evaluated through a spectrophotometric assay, which was modified from the methods described by Thring et al. (2009) [21]. Initially, an MMP-1 solution at a concentration of 0.1 units/mL was prepared, comprising MMP-1 sourced from *C. histolyticum*, along with 400 mM NaCl, 10 mM CaCl_2_, and 50 mM tricine buffer pH 7.5. Subsequently, 20 µL of the sample solution was combined with 20 µL of the MMP-1 solution and 80 µL of Tricine buffer pH 7.5. After the resulting mixture was incubated for 15 min at ambient temperature, 100 µL of a substrate solution consisting of 0.8 mM FALGPA in tricine buffer with pH 7.5 was introduced to initiate the enzymatic reaction. Immediately, the absorbance was measured kinetically at 340 nm for a duration of 20 min using a multimode microplate reader (BMG Labtech GmbH, Ortenberg, Germany). The findings were then expressed as the percentage of MMP-1 inhibition, calculated according to the subsequent equation:

MMP-1 inhibition (%) = [(a − b)/a] × 100,(3)

where a is the reaction rate of the combination without a sample, while b is the reaction rate of the combination with a sample. Various concentrations of each extract were evaluated, and their MMP-1 inhibitions were plotted against their logarithmic concentrations to generate dose–response curves. The IC_50_ values were calculated from the curves using GraphPad Prism (version 8.0.2, GraphPad Software, Boston, MA, USA). EGCG acid was used as the positive control. The analysis was performed in triplicate.

4. Elastase Inhibition

The capability of the extracts derived from the adult *V. coerulea* plant and its protocorms to inhibit elastase activities was evaluated through a spectrophotometric assay, which was modified from the methods described by Thring et al. (2009) [21]. Initially, an elastase solution at a concentration of 0.03 units/mL was prepared, comprising elastase sourced from porcine pancreas, along with 0.2 M Tris-HCl buffer pH 8.0. Subsequently, 10 µL of the sample solution was combined with 40 µL of the elastase solution. After the resulting mixture was incubated for 15 min at ambient temperature, 100 µL of 1.6 mM AAAVPN in tris HCl buffer pH 8.0 was introduced to initiate the enzymatic reaction. Immediately, the absorbance was measured kinetically at 410 nm for a duration of 20 min using a multimode microplate reader (BMG Labtech GmbH, Ortenberg, Germany). The findings were then expressed as the percentage of elastase inhibition, calculated according to the subsequent equation:

Elastase inhibition (%) = [(a − b)/a] × 100,(4)

where a is the reaction rate of the combination without a sample, while b is the reaction rate of the combination with a sample. Various concentrations of each extract were evaluated, and their elastase inhibitions were plotted against their logarithmic concentrations to generate dose–response curves. The IC_50_ values were calculated from the curves using GraphPad Prism (version 8.0.2, GraphPad Software, Boston, MA, USA). EGCG acid was used as the positive control. The analysis was performed in triplicate.

#### 2.6.3. Anti-Tyrosinase Activities

The capability of the extracts derived from the adult *V. coerulea* plant and its protocorms to inhibit the tyrosinase activities was evaluated through a spectrophotometric assay, which was modified from the methods described by Saeio et al. (2011) [20]. Initially, a tyrosinase solution at a concentration of 250 unit/mL was prepared, comprising tyrosinase sourced from mushroom, along with 20 mM phosphate buffer with a pH of 6.8. Subsequently, 10 µL of the sample solution was combined with 30 µL of the tyrosinase solution. After the resulting mixture was incubated for 10 min at ambient temperature, 100 μL of 12 mM L-DOPA was added and then incubated for an additional 30 min. The absorbance was subsequently measured at 510 nm using a multimode microplate reader (BMG Labtech GmbH, Ortenberg, Germany). The findings were then expressed as the percentage of tyrosinase inhibition, calculated according to the subsequent equation:

Tyrosinase inhibition (%) = [(a − b)/a] × 100,(5)

where a is the UV absorbance of the combination without a sample, while b is the UV absorbance of the combination with a sample. Various concentrations of each extract were evaluated, and their tyrosinase inhibitions were plotted against their logarithmic concentrations to generate dose–response curves. The IC_50_ values were calculated from the curves using GraphPad Prism (version 8.0.2, GraphPad Software, Boston, MA, USA). Kojic acid was used as the positive control. The analysis was performed in triplicate.

#### 2.6.4. Anti-Inflammatory Activities

The capability of the extracts derived from the adult *V. coerulea* plant and its protocorms to inhibit the inflammatory cytokines expression, including interleukin-6 (IL-6) and tumor necrosis factor-α (TNF-α), was evaluated through a real-time polymerase chain reaction quantification (qPCR), which was modified from the methods described by Lee et al. (2019) [22]. Prior to qPCR analysis, the viability of MC3T3-E1 cells was evaluated using the 3-[4,5-dimethylthiazol-2-yl]-2,5 diphenyl tetrazolium bromide (MTT) assay. In brief, 2 × 10^4^ cells per well of MC3T3-E1 cells were seeded in a 96-well plate and incubated at a temperature of 37 °C with 5% CO_2_ and 90% humidity for a duration of 24 h. Subsequently, *V. coerulea* adult plant and protocorm extracts were administered to the MC3T3-E1 cells and incubated for 48 h. After the designated time elapsed, 100 µL of media was aspirated from each well, and 25 µL of 5 mg/mL of MTT solution was introduced into each well. After another incubation for a period of 4 h, all media were removed by inverting the 96-well plate, and 200 µL of DMSO was added to dissolve the formazan crystals. Finally, the 96-well plate was subjected to spectrophotometric analysis at 595 nm using a multimode microplate reader (BMG Labtech GmbH, Ortenberg, Germany).

To assess the inhibition of IL-6 and TNF-α by the extracts obtained from the adult *V. coerulea* plant and its protocorms, LPS was used to induce the inflammatory process in MC3T3-E1 cells. Following the treatment of each sample, the cells from each group were harvested, and total RNA extraction was performed using TRIZOL^®^ reagent per the manufacturer’s guidelines. To eliminate genomic DNA contamination, the isolated total RNA was treated with TURBO™ DNase according to the manufacturer’s instructions. The resulting purified total RNA was employed for cDNA synthesis using the iScript™ cDNA Synthesis Kit per the manufacturer’s instructions. Quantification of qPCR was carried out using the CFX Connect Thermal Cycler (Bio-Rad Laboratories Ltd., Hemel Hempstead, UK). Each PCR reaction comprised 7.5 μL of SYBR^®^ green, 0.5 μL of cDNA, specific forward and reverse primers at concentrations ranging from 0.1 to 0.4 μM, and the volume was adjusted to 10 μL with sterile distilled water. The thermal cycling conditions encompassed an initial step of 15 min at 95 °C, succeeded by 40 cycles consisting of 10 s at 95 °C, 15 s at 58 °C, and 20 s at 72 °C. Comprehensive primer details are presented in Table 1. The obtained real-time data were analyzed using CFX Maestro software version 2.3 (Bio-Rad Laboratories Ltd., Hemel Hempstead, UK). The expression of target genes (IL-6 and TNF-α) was normalized against the expression level of β-actin. The findings were then expressed as the percentage of IL-6 or TNF-α inhibition, calculated according to the subsequent equations:

IL-6 inhibition (%) = [(a − b)/a] × 100,(6)

TNF-α inhibition (%) = [(a − b)/a] × 100,(7)

where a is the expression level of cytokines without the sample, while b is the expression level of cytokines with the sample. Dexamethasone was used as the positive control. The analysis was performed in triplicate.

### 2.7. Determination of Irritation Potency of V. coerulea Adult Plant and Protocorm Extracts by Hen’s Egg-Chorioallantoic Membrane (HET-CAM) Test

The irritation potency of the extracts derived from the adult *V. coerulea* plant and its protocorms was evaluated through a HET-CAM test, which was previously described by Luepke and Kemper (1986) [23]. The fertilized black hen eggs from the Pradu Hang Dam Chiang Mai breed aged between 7 and 9 days were used in the HET-CAM test. As the embryos were in the early stages of development, ethical approval was not required. Typically, hen eggs require 21 days of incubation, and the fertilized eggs aged between 7 to 9 days, which was less than halfway through their incubation cycle, were not considered animals. Prior to the test, the eggshell above the air cell was cracked open, and the inner membrane was moistened with a normal saline solution. Subsequently, 30 µL of each sample solution was applied to the CAM, and the irritation signs were observed under a stereomicroscope (Olympus, Tokyo, Japan) in terms of vascular coagulation, lysis, and hemorrhage within 5 min and again after 60 min. The duration until the first appearance of each irritation sign was meticulously monitored and recorded in seconds, which enabled the subsequent computation of the irritation score (IS) using the following equation:

IS = [(301 − t(h))/300] × 5 + [(301 − t(l))/300] × 7 + [(301 − t(c))/300] × 9,(8)

where t(h) is the time of the first appearance of hemorrhage in seconds, t(l) is the time of the first appearance of vascular lysis in seconds, and t(c) is the first appearance of vascular coagulation in seconds. The IS was then divided into the following categories: 0–0.9 for no irritation, 1.0–4.9 for mild irritation, 5.0–8.9 for moderate irritation, and 9.0–21.0 for severe irritation [24]. A 1% *w*/*v* sodium lauryl sulfate solution served as the positive control, while a normal saline solution (0.9% *w*/*v* NaCl aqueous solution) acted as the negative control. The analysis was performed in triplicate.

### 2.8. Statistical Analysis

The data were expressed as the mean and standard deviation (SD). Differences among samples were assessed using one-way ANOVA conducted in GraphPad Prism (version 8.0.2, GraphPad Software, Boston, MA, USA), followed by Tukey’s post hoc test. Significance was determined at a *p*-value < 0.05.

## 3. Results and Discussion

### 3.1. Adult V. coerulea Plants and Protocorms

**Figure 1:**
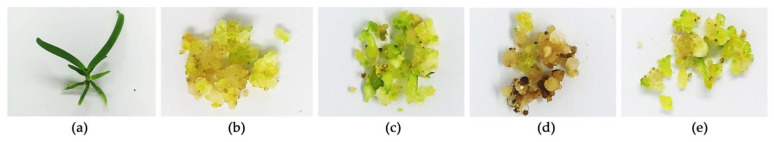
The external appearance of the eight-month-old *V. coerulea* (**a**) along with the four-week-old protocorm without elicitor (**b**), with 3 µM 6-benzylaminopurine (BA) (**c**), 10 µM methyl jasmonate (MeJA) (**d**), and 2 µM chitosan (**e**).

Adult plants and protocorms of *V. coerulea* are shown in Figure 1. The eight-month-old *V. coerulea* plant had robust, leathery, channeled leaves that are closely packed, strap-shaped, and green in color, with a truncate apex and considerable length. Additionally, the adult *V. coerulea* had thick green stems and green roots. In contrast, *V. coerulea* protocorms, originating from the orchid embryo before its transformation into a plantlet, were observed as spherical entities with globular structures. Various colors of protocorms were observed following treatment with different elicitors. The control protocorms without elicitor treatment were yellowish, as shown in Figure 1b.

However, after being treated with BA and chitosan, the protocorms tended to develop a greener color, whereas MeJA produced a distinct darker coloration. The findings were likely attributed to the ability of BA to enhance green pigmentation by inhibiting the activity of chlorophyll degradation enzymes, thus resulting in a delayed decline [25]. In addition, BA has been reported to enhance chloroplast formation and photosynthesis, leading to increased chlorophyll production, which gives plants their green color [26]. Therefore, protocorms of *V. coerulea* treated with BA were found to be greener than the control protocorms, as shown in Figure 1c. Comparable results were noted in a study involving broccoli where florets treated with BA demonstrated elevated chlorophyll levels [25]. Furthermore, BA was classified as a cytokinin belonging to a specific group of plant hormones with pivotal roles in diverse aspects of plant growth and development, involving various regulation processes, e.g., cell division, shoot and root development, control of lateral bud growth, influence on seed germination, and the delay of aging [27].

On the other hand, MeJA has been identified as a potent elicitor that enhances plant resilience in challenging environments by activating defense mechanisms through the induction of various enzymes, including phenyl ammonia lyase, catalase, chalcone isomerase, stilbene synthase, and anthocyanin synthase [28,29,30,31]. MeJA influenced various plant processes, including seed germination, root growth, gravitropism, and overall plant development [32,33]. The darker protocorms observed in the current study of *V. coerulea*, as shown in Figure 1d, are consistent with previous findings by Donnez et al. (2011) that exhibited similar observations in a study on grapevine rootstock 41B where MeJA elicitation induced a shift from yellow to dark brown cell color [34].

Chitosan, a natural polysaccharide, serves as an elicitor due to its inherent ability to instigate resistance against adverse stress conditions, enhance photosynthesis rates, facilitate plant growth, and prompt plant resistance responses to both biotic and abiotic stressors [35]. As chitosan enhanced photosynthesis rates, resulting in increased chlorophyll production and the characteristic green coloration [36], the protocorms of *V. coerulea* treated with chitosan were found to be greener in color compared with the control, as shown in Figure 1e. However, although different characteristics of protocorms were found after treatment with various elicitors, these visual characteristics do not provide insight into internal composition, highlighting the need for further assessment of chemical content to gain a comprehensive understanding of the underlying factors.

### 3.2. Chemical Compositions of V. coerulea Plant and Protocorm Extracts

**Figure 2:**
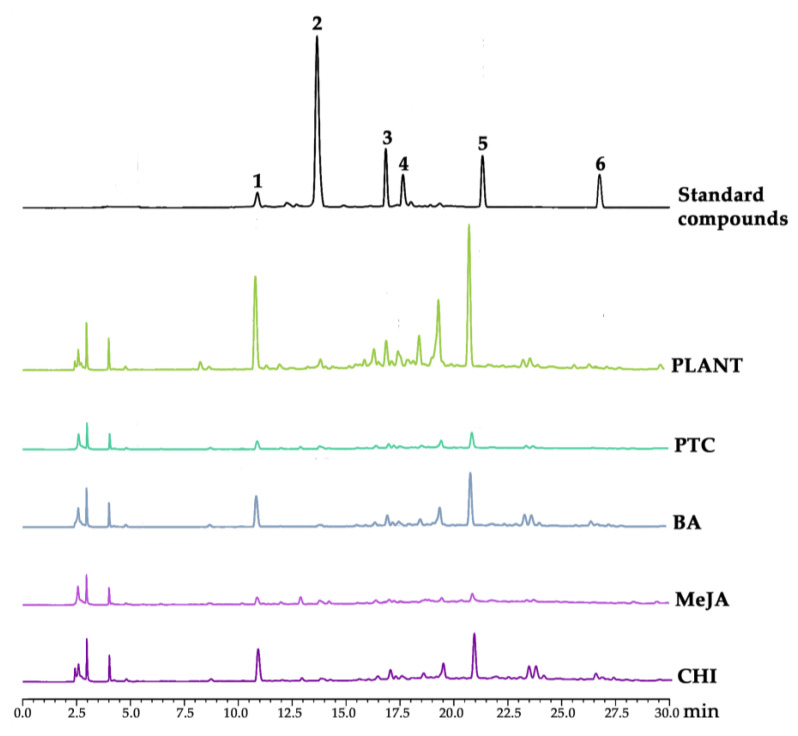
HPLC chromatograms of standard compounds, including catechin (1), syringic acid (2), rutin (3), ellagic acid (4), rosmarinic acid (5), quercetin (6), and the methanolic extracts of eight-month-old *V. coerulea* (PLANT), four-week-old *V. coerulea* protocorm without elicitor (PTC) or with 3 µM 6-benzylaminopurine (BA), 10 µM methyl jasmonate (MeJA), and 2 µM chitosan (CHI).

The methanolic extracts from both *V. coerulea* plant and protocorms were examined for their chemical compositions, and their HPLC chromatograms are shown in Figure 2. Various phytochemical constituents were detected in the *V. coerulea* extracts, including catechin, syringic acid, rutin, ellagic acid, rosmarinic acid, and quercetin, with retention times of 10.921, 13.683, 16.869, 17.665, 21.347, and 26.769 min, respectively. However, there was a prominent unidentified peak around the retention time of 20 min. The unidentified peak may potentially represent a derivative of the detected phytochemical compounds, or it could be a derivative of eucomic acid or phenanthrene, both of which have been previously identified as bioactive compounds in *V. coerulea* [3]. Furthermore, the presence of other unidentified HPLC peaks highlighted the need for a thorough investigation and further definitive identification. Nevertheless, the current study reported that the methanolic extract derived from the *V. coerulea* adult plant exhibited significantly higher levels of phytochemical constituents compared with unelicited protocorms, which showed minimal presence of these compounds. Elicitations were observed to elevate the concentration of all phytochemical constituents, except in the case of MeJA. However, the adult plants contained the highest levels of most identified phytochemical constituents. The likely explanation could stem from the maturation process as these compounds are typically abundant in stems, leaves, or pseudobulbs [37,38,39]. Consequently, younger cells may not accumulate them at the same levels as older plants. Although the amounts of major chemical compounds tested in this study in elicited protocorms were not as high as in adult plants, they still increased compared with unelicited cells.

Additionally, some compounds, such as syringic acid and quercin, exhibited comparable or higher amounts in elicited cells compared with adult plants, as listed in Table 2. It was observed that syringic acid was detected at higher levels in both elicited and unelicited protocorms, particularly when elicited with MeJA. This corresponded to the defense mechanism of plants as syringic acid is recognized for its involvement in lignification, which is a process by which plants reinforce their cell walls, particularly in response to stress or damage, to provide structural support and enhance resistance against pathogens or environmental stressors [40]. Also, a previous study reported the observation of syringic acid accumulation in soybean plants under water stress conditions, emphasizing its role in promoting cell division [41,42].

On the other hand, the levels of rosmarinic acid in protocorms elicited with BA and chitosan were equivalent to those found in an adult plant. The findings were in line with a previous study reporting that undifferentiated cultures, i.e., callus and suspension cultures, have been known to frequently yield higher quantities of rosmarinic acid compared with intact plants [43]. Chitosan has been reported to enhance the production of various secondary metabolites in many plants, e.g., scopolamine and hyoscyamine in *Brugmansia candida* [44], hyoscyamine in *Hyoscyamus muticus* [45], β-1,3-glucan in *Petroselinum crispum* [46], hernandulcin in *Lippia dulcis* [47], diosgenin in *Trigonella foenumgraecum* L. [48], etc. In addition, the application of chitosan lactate through foliar spray resulted in an elevated accumulation of rosmarinic acid since chitosan served as an elicitor through enhancing defense molecules and secondary metabolites in ornamental plants during stressful conditions [49]. Quercetin was also found to be enhanced after the treatment with chitosan in the current study.

Among various phytochemical compositions, rutin was observed to be present in higher concentrations compared with the other identified compounds. Despite exhibiting a smaller peak compared with other compounds, particularly catechin, rutin was found in higher quantities. The likely explanation could be attributed to the quantitative determination of each individual compound based on their respective standard curves. The varying maximum absorbance of each substance caused different slopes in the standard curves, resulting in distinct formulations for the quantitative calculations. However, since HPLC analysis was performed at a fixed wavelength of 280 nm, the quantity of each substance assessed against a standard curve did not directly correspond to the peak height or area but depended on the calculations derived from the standard curve. Rutin, which has a maximum absorbance at 360 nm [50], exhibited a smaller peak than catechin, which has absorbance maxima at both 280 nm and 440 nm [51] because the analysis was performed at 280 nm. At this wavelength, catechin absorbed more strongly due to its absorbance maximum at 280 nm, resulting in a higher peak in the chromatogram. In contrast, rutin did not absorb as effectively at 280 nm, leading to a smaller peak despite potentially higher concentrations. This discrepancy arose because the detection sensitivity at 280 nm was greater for catechin than for rutin. Consequently, although rutin was presented in higher quantities, its peak appeared smaller due to its lower absorbance at the selected analysis wavelength. On the other hand, despite the lower content of rutin observed in *V. coerulea* protocorms compared with that in the adult plant, it was noted that both BA and chitosan application led to an obvious increase in rutin levels within the extract, highlighting the potential of these elicitors to positively influence rutin accumulation in the protocorms.

### 3.3. Antioxidant Activities of V. coerulea Plant and Protocorm Extracts

**Figure 3:**
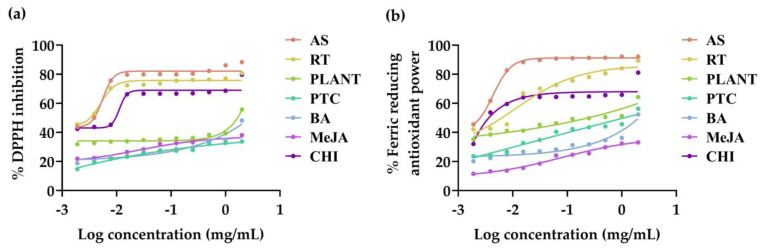
Dose–response curves against DPPH inhibition (**a**) and ferric reducing antioxidant power (**b**) of ascorbic acid (AS), rutin (RT), and the methanolic extracts of eight-month-old *V. coerulea* (PLANT), four-week-old *V. coerulea* protocorm without elicitor (PTC) or with 3 µM 6-benzylaminopurine (BA), 10 µM methyl jasmonate (MeJA), and 2 µM chitosan (CHI).

Antioxidant activities of *V. coerulea* plant and protocorm extracts were evaluated in terms of DPPH inhibition and ferric reducing antioxidant power, with the dose–response curve shown in Figure 3 and the corresponding IC_50_ values presented in Table 3. Rutin, which was a major identified constituent, was also evaluated. The entire sample was found to scavenge DPPH^•^ radicals and reduce ferric in a dose-dependent manner. Although the DPPH assay was used to evaluate the radical scavenging ability, and the FRAP assay exhibited the ferric reducing ability of the antioxidants, both assays showed consistent results. Ascorbic acid, the strongest reductant and radical scavenger [52], exhibited the most potent DPPH^•^ radical scavenging and ferric reducing properties. The findings could be explained by the fact that ascorbic acid served as a donor of single hydrogen atoms and frequently underwent a transition between its fully reduced state (AH^−^) and the radical anion form referred to as monodehydroascorbate (A^•−^) [53]. In addition, rutin was found to be a phytochemical compound responsible for the antioxidant activities of *V. coerulea* extracts, particularly in terms of DPPH radical scavenging activity. This is evident as rutin exhibited DPPH^•^ radical scavenging activities comparable to those of ascorbic acid. The findings were in line with previous reports, which clearly indicated that rutin exhibited strong DPPH^•^ radical scavenging activity and powerful antioxidant capacity against various antioxidant systems in vitro, with the capacity being concentration-dependent [54]. Among the *V. coerulea* extracts, the protocorm treated with chitosan exhibited the highest ferric reducing ability, which was comparable to that of ascorbic acid. The explanation could be that chitosan, which was reported to function as a plant growth promoter in various plants, particularly orchids, elicited defense mechanisms and participated in the signaling pathway for the biosynthesis of phenolics [55].

### 3.4. Anti-Skin Aging Activities of V. coerulea Plant and Protocorm Extracts

**Figure 4:**
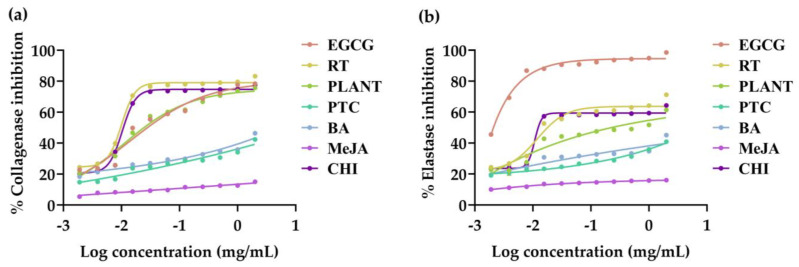
Dose–response curves against collagenase inhibition (**a**) and elastase inhibition (**b**) of epigallocatechin gallate (EGCG), rutin (RT), and the methanolic extracts of eight-month-old *V. coerulea* (PLANT), four-week-old *V. coerulea* protocorm without elicitor (PTC) or with 3 µM 6-benzylaminopurine (BA), 10 µM methyl jasmonate (MeJA), and 2 µM chitosan (CHI).

Anti-skin aging activities of *V. coerulea* plant and protocorm extracts were evaluated in terms of collagenase and elastase inhibition, with the dose–response curve shown in Figure 4 and the corresponding IC_50_ values presented in Table 3. Collagen serves as the principal protein in the dermal layer, forming the primary component of the skin’s dermis. Its decline is a contributing factor to skin aging [56]. While its fundamental function involves maintaining skin strength and elasticity, the aging process may introduce changes in the control of collagen degradation and synergy. Collagenase, an enzyme implicated in collagen breakdown, plays a significant role in this process [57]. Therefore, the potential to inhibit the activity of this enzyme holds promise for safeguarding against skin aging, presenting a valuable opportunity within the cosmetic industry. The extracts from the *V. coerulea* plant and protocorms held great promise in reducing skin wrinkles, attributed to their notable ability to inhibit collagenase. Among various *V. coerulea* extracts, the protocorm treated with chitosan was found to be the most potent collagenase inhibitor, with an IC_50_ value of 7.8 ± 3.5 µg/mL, comparable to rutin, a major identified constituent (IC_50_ value of 7.6 ± 3.4 µg/mL). Interestingly, its collagenase inhibition was superior to that of EGCG, a well-known anti-collagenase, which had the IC_50_ value of 16.9 ± 3.2 µg/mL. It was also found that the extract from *V. coerulea* adult plant had comparable collagenase inhibitory activities to EGCG. Therefore, it was reported that *V. coerulea* extract held a significant promise as a candidate for collagenase inhibition, with the protocorm treatment showing exceptional potency, even surpassing that of EGCG, a positive control.

Aside from collagenase, there are two distinct types of elastases: neutrophil elastase and skin fibroblast elastase. These enzymes hold primary responsibility for the degradation of elastic fibers associated with the development of wrinkles [58]. Consequently, the integration of ingredients capable of inhibiting elastase activity becomes a viable option for cosmetic formulations aimed at counteracting skin aging and wrinkles. While the results of this study reveal unequaled anti-elastase efficacy in *V. coerulea* extracts compared with EGCG, promising inhibitory activities were detected in the adult plant, protocorm treated with chitosan and rutin. Therefore, rutin could be concluded as a major compound responsible for the anti-aging activities of *V. coerulea* extracts, exhibiting notable inhibitory effects against both collagenase and elastase.

### 3.5. Anti-Tyrosinase Activities of V. coerulea Plant and Protocorm Extracts

**Figure 5:**
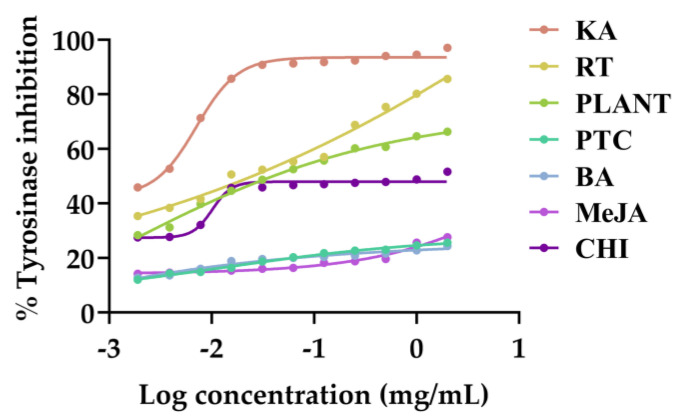
Dose–response curves against tyrosinase inhibition of kojic acid (KA), rutin (RT), and the methanolic extracts of eight-month-old *V. coerulea* (PLANT), four-week-old *V. coerulea* protocorm without elicitor (PTC) or with 3 µM 6-benzylaminopurine (BA), 10 µM methyl jasmonate (MeJA), and 2 µM chitosan (CHI).

Anti-tyrosinase activities of *V. coerulea* plant and protocorm extracts were evaluated, with their dose–response curves shown in Figure 5 with the corresponding IC_50_ values presented in Table 3. As tyrosinase catalyzes the conversion of L-tyrosine into 3,4-dihydroxyphenylalanine (DOPA), subsequent to the transformation of DOPA into DOPA quinone and, ultimately, melanin pigment formation, inhibiting tyrosinase becomes crucial for investigating the whitening effects of the extracts [59]. *V. coerulea* extracts were found to exhibit promising anti-tyrosinase activities, particularly the extract from the adult plant (IC_50_ value of 10.8 ± 3.5 µg/mL) and protocorm treated with chitosan plant (IC_50_ value of 8.1 ± 2.8 µg/mL). Rutin was found to be responsible for anti-tyrosinase activities as its anti-tyrosinase effect had the IC_50_ value of 7.7 ± 3.1, which was comparable to those promising extracts. However, the inhibitory effects were not as potent as kojic acid, a well-known whitening agent. Therefore the *V. coerulea* extracts did not demonstrate significant effectiveness in countering skin pigmentation.

### 3.6. Anti-Inflammatory Activities of V. coerulea Plant and Protocorm Extracts

**Figure 6:**
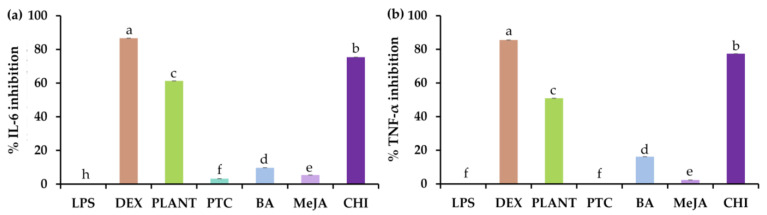
Inhibitory activities against interleukin-6 (IL-6) (**a**) and tumor necrosis factor-α (TNF-α) (**b**) of MC3T3-E1 cells treated with lipopolysaccharide (LPS) and along with dexamethasone (DEX), and the methanolic extracts of eight-month-old *V. coerulea* (PLANT), four-week-old *V. coerulea* protocorm without elicitor (PTC), with 3 µM 6-benzylaminopurine (BA), 10 µM methyl jasmonate (MeJA), and 2 µM chitosan (CHI). Different letters denote significant differences among the extracts (*p* < 0.05).

The anti-inflammatory activities of *V. coerulea* plant and protocorm extracts were evaluated, and their inhibitory activities are shown in Figure 6, which presents the expression of important inflammatory mediators, IL-6 and TNF-α, through qPCR, with normalization relative to the expression of β-actin mRNA. Inflammation constitutes a condition characterized by localized increments in leukocyte counts and an intricate interplay of diverse mediator molecules. Mouse calvaria-derived osteoblast-like cells (MC3T3-E1) have the inherent capability to generate pro-inflammatory cytokines, including IL-6 and TNF-α [60]. Consequently, these cells serve as a suitable model for assessing the anti-inflammatory potential of the *V. coerulea* extracts. The genes encoding pro-inflammatory mediators are robustly activated during inflammation, driving its onset and persistence. IL-6, a pleiotropic cytokine boasting an extensive array of biological functions, is upregulated during inflammatory processes and can contribute to malignancy in the skin [61]. Likewise, TNF-α served as a signaling molecule for immune cells, orchestrating inflammatory responses and exerting various pro-inflammatory effects on the skin [62]. Current findings showed that LPS, a renowned pro-inflammatory cytokine inducer [63,64], effectively induced IL-6 and TNF-α expression. LPS-treated MC3T3-E1 cells noticeably amplified the expression of IL-6 and TNF-α, aligning with their pro-inflammatory nature. Interestingly, the *V. coerulea* extract from protocorm treated with chitosan showed strong anti-inflammatory properties, suppressing IL-6 and TNF-α expression by 75.38 ± 0.02% and 77.40 ± 0.02%, respectively. Although its IL-6 and TNF-α inhibitions were not potent as dexamethasone, a well-known nonsteroidal anti-inflammatory drug (NSAID), it was more potent than the extract from the adult *V. coerulea* plant. Hence, it was found that the extract derived from chitosan-treated *V. coerulea* protocorms minished the expression of genes associated with inflammatory mediators and that both IL-6 and TNF-α, implied the presence of anti-inflammatory properties within the extracts. This suggests their potential application as effective ingredients in cosmetics. Considering the pivotal roles of both IL-6 and TNF-α in skin physiology, these extracts hold promise for enhancing skin health and overall well-being. Therefore, protocorms were found to serve to shorten cultivation durations, thereby conserving both energy and land resources while concurrently enhancing the production efficacy of bioactive compounds with greater potency.

The anti-inflammatory properties of *V. coerulea* extracts aligned with other biological activities pertinent to cosmeceutical applications, including antioxidant, anti-skin aging, and anti-tyrosinase effects. Remarkably, the extract from *V. coerulea* protocorm treated with chitosan displayed the highest potency across all these areas. Therefore, this extract emerged as a promising candidate for cosmeceutical formulations, offering a comprehensive array of beneficial effects.

### 3.7. Irritation Potency of V. coerulea Protocorm Extracts

**Figure 7:**
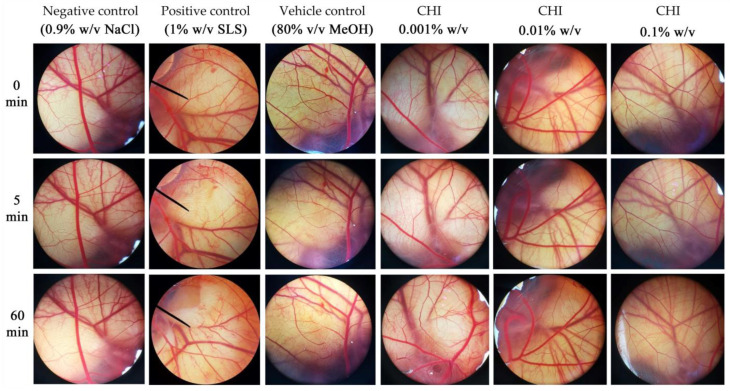
The photographs illustrating the impacts of various substances, including negative control (0.9% *w*/*v* NaCl), positive control (1% *w*/*v* SLS), vehicle control (80% *v*/*v* MeOH), and the extract of four-week-old *V. coerulea* protocorm treated with 2 µM chitosan (CHI) at concentrations of 0.001, 0.01, and 0.01% *w*/*v*, that were applied to the chorioallantoic membrane before treatment (0 min), after a 5 min interval, and at the 60 min endpoint.

As the extract from *V. coerulea* protocorm treated with chitosan exhibited significant potential for use as an active component in further cosmeceutical formulation development, addressing the potential concern of irritation becomes crucial when considering the utilization of novel active cosmeceutical ingredients. The irritation potency of the extract from *V. coerulea* protocorm treated with chitosan was evaluated by the HET-CAM test, which has gained widespread acceptance as an alternative method to the in vivo irritation test. In recent years, it has also found utility as a preliminary screening assay prior to in vivo studies, aiding in the assessment of the irritant potential of substances when in contact with membranes and skin [65]. The outcomes pertaining to irritant potential are depicted in Figure 7 and summarized in Table 4. Remarkably, the IS for the positive control, 1% *w*/*v* SLS, was calculated at 15.13 ± 0.3. This notably high IS value unequivocally categorizes substances of this nature as extreme irritants. In contrast, as anticipated, the negative control samples containing 0.9% *w*/*v* NaCl showed no signs of irritant reactions. Furthermore, it is noteworthy that the vehicle control group, consisting of an 80% *v*/*v* MeOH solution, exhibited severe irritant effects, suggesting a potential lack of safety for topical application. Interestingly, treatments involving the extract of *V. coerulea* protocorm treated with chitosan yielded nonirritant effects. The findings indicate the potential suitability for topical application without causing irritation of the extract of *V. coerulea* protocorm treated with chitosan. Although HET-CAM was used as the in vitro irritation study in the current research, further dermatological tests are recommended to evaluate the safety for cosmetic use.

## 4. Conclusions

The integration of cell culture and elicitation techniques has significantly enhanced the production of valuable bioactive compounds from *V. coerulea,* specifically for use in the cosmeceutical field. The findings from this study report on the potential of *V. coerulea* as a source of chemical compounds capable of a variety of biological activities, with rutin being the most abundant identified component. Although the content of identified bioactive compounds in each *V. coerulea* protocorm extract did not reach the levels of the adult plant, the biological activities were more potent. Chitosan-treated *V. coerulea* protocorms showed remarkable biological properties, including potent collagenase inhibition, anti-tyrosinase activities, and strong anti-inflammatory effects. Importantly, the elicited protocorm extracts demonstrated safety and nonirritating characteristics, making them promising candidates for cosmeceutical applications. Additionally, a future promising alternative green extraction method using green solvents, such as water, aligning with sustainable and environmentally friendly practices, is suggested. Following this, the freeze-drying process would make the extract easier to incorporate into cosmetic products. Notably, utilizing protocorms offers advantages over adult plants due to their shorter duration of development, which significantly enhances the sustainability of cultivation practices by minimizing resource consumption. Furthermore, the implementation of protocorm and elicitation techniques for the propagation of *V. coerulea* could replace the dependency on rare plant specimens. Further investigation into the stability of the extracts under various conditions and their compatibility with common cosmetic additives is recommended.

## Figures and Tables

**Table 1:** Primers used for qPCR [22].

Gene	Primer	Sequence (5′-3′)
IL-6	Forward	GAGACTTCCATCCAGTTGCC
	Reverse	TACTCCAGAAGACCAGAGG
TNF-α	Forward	GGGACAGTGACCTGGACTGT
	Reverse	GCAGAGGTTCAGTGATGTAG
β-Actin	Forward	TGGATGGCTACGTACATGGCTGGG
	Reverse	TTCTTTGCAGCTCCTTCGTTGCCG

**Table 2:** Chemical compositions of the methanolic extracts of *V. coerulea* adult plant and protocorms treated with various elicitors.

Standard Compounds	Retention Time (min)	Chemical Compositions (mg/g Extract)				
		PLANT	PTC	BA	MeJA	CHI
Catechin	10.920	17.5 ± 0.0 ^a^	1.5 ± 0.0 ^c^	5.7 ± 0.0 ^b^	1.3 ± 0.0 ^c^	5.8 ± 0.0 ^b^
Syringic acid	13.671	0.1 ± 0.0 ^d^	1.4 ± 0.0 ^b^	0.9 ± 0.0 ^c^	1.7 ± 0.0 ^a^	1.3 ± 0.0 ^b^
Rutin	16.880	33.4 ± 0.0 ^a^	5.0 ± 0.0 ^c^	12.8 ± 0.0 ^b^	4.6 ± 0.0 ^d^	12.1 ± 0.0 ^b^
Ellagic acid	17.605	3.9 ± 0.0 ^a^	0.4 ± 0.0 ^c^	1.0 ± 0.0 ^b^	0.2 ± 0.0 ^d^	0.9 ± 0.0 ^b^
Rosmarinic acid	21.346	0.1 ± 0.0 ^a^	0.0 ± 0.0 ^b^	0.1 ± 0.0 ^a^	0.0 ± 0.0 ^b^	0.1 ± 0.0 ^a^
Quercetin	26.769	0.2 ± 0.0 ^b^	0.0 ± 0.0 ^c^	0.2 ± 0.0 ^b^	0.0 ± 0.0 ^c^	0.3 ± 0.0 ^a^

NOTE: PLANT = methanolic extracts of eight-month-old *V. coerulea*; PTC = methanolic extracts of *V. coerulea* protocorm without elicitor; BA = methanolic extracts of *V. coerulea* protocorm treated with 3 µM 6-benzylaminopurine; MeJA = methanolic extracts of *V. coerulea* protocorm treated with 10 µM methyl jasmonate; CHI = methanolic extracts of *V. coerulea* protocorm treated with 2 µM chitosan. Different letters (^a^, ^b^, ^c^, and ^d^) denote significant differences among the extracts (*p* < 0.05).

**Table 3:** Biological activities related to cosmeceutical properties of *V. coerulea* plant and protocorm extracts.

Samples	IC_50_ (µg/mL)				
	DPPH Inhibition	Ferric Reducing Capacity	Collagenase Inhibition	Elastase Inhibition	Tyrosinase Inhibition
AS	3.5 ± 2.9 ^a^	2.4 ± 0.7 ^a^	N/A	N/A	N/A
EGCG	N/A	N/A	16.9 ± 3.2 ^b^	0.1 ± 0.0 ^a^	N/A
KA	N/A	N/A	N/A	N/A	2.8 ± 0.6 ^a^
RT	3.2 ± 2.7 ^a^	12.9 ± 2.0 ^b^	7.6 ± 3.4 ^a^	10.5 ± 1.9 ^b^	7.7 ± 3.1 ^b^
PLANT	ND	25.8 ± 4.7 ^c^	12.3 ± 2.3 ^b^	11.3 ± 7.9 ^b^	10.8 ± 3.5 ^b^
PTC	ND	27.3 ± 5.0 ^c^	27.3 ± 8.6 ^c^	ND	ND
BA	ND	60.3 ± 5.0 ^d^	24.5 ± 3.2 ^c^	ND	ND
MeJA	ND	73.8 ± 8.8 ^e^	ND	ND	ND
CHI	9.3 ± 2.7 ^b^	2.1 ± 0.1 ^a^	7.8 ± 3.5 ^a^	8.4 ± 3.3 ^b^	8.1 ± 2.8 ^b^

NOTE: AS = ascorbic acid; EGCG = epigallocatechin gallate; KA = kojic acid; RT = rutin; PLANT = methanolic extracts of eight-month-old *V. coerulea*; PTC = methanolic extracts of *V. coerulea* protocorm without elicitor; BA = methanolic extracts of *V. coerulea* protocorm treated with 3 µM 6-benzylaminopurine; MeJA = methanolic extracts of *V. coerulea* protocorm treated with 10 µM methyl jasmonate; CHI = methanolic extracts of *V. coerulea* protocorm treated with 2 µM chitosan; N/A = not available; ND = not determined due to low potency. Different letters (^a^, ^b^, ^c^, ^d^, and ^e^) denote significant differences among the extracts (*p* < 0.05).

**Table 4:** Irritation score (IS) and irritancy classification in HET-CAM test.

Treatment	Irritation Score (IS)	Irritation Level
Negative control (0.9% *w*/*v* NaCl)	0.0 ± 0.0	No irritation
Positive control (1% *w*/*v* SLS)	15.13 ± 0.3	Severe irritation
Vehicle control (80% *v*/*v* MeOH)	9.1 ± 0.6	Severe irritation
CHI 0.001% *w*/*v*	0.0 ± 0.0	No irritation
CHI 0.01% *w*/*v*	0.0 ± 0.0	No irritation
CHI 0.1% *w*/*v*	0.46 ± 0.01	No irritation

## Data Availability

Data are available upon request.
